# Complete Genome Sequence of a UL96 Mutant Cytomegalovirus Towne-BAC (Bacterial Artificial Chromosome) Isolate Passaged in Fibroblasts To Allow Accumulation of Compensatory Mutations

**DOI:** 10.1128/genomeA.00901-13

**Published:** 2013-10-24

**Authors:** Teal M. Brechtel, Molly Tyner, Ritesh Tandon

**Affiliations:** Department of Microbiology, University of Mississippi Medical Center, Jackson, Mississippi, USA

## Abstract

Here, we present the complete genome sequence of a cytomegalovirus Towne-BAC (bacterial artificial chromosome) isolate that we first genetically engineered to mutate the UL96 gene and then serially passaged in human fibroblasts to allow for the accumulation of compensatory mutations. A total of 17 single-base substitutions were discovered in the passaged genome compared to the reference sequence (KF493877).

## GENOME ANNOUNCEMENT

Slowly replicating mutant viruses can be passaged in cell culture to allow for the accumulation of compensatory mutations. This passaging often results in increased virus growth rates over a period of time (1–5). We engineered a cluster mutation in the human cytomegalovirus Towne-BAC (bacterial artificial chromosome) genome (6), replacing the UL96 C-terminal _122_EVDDAV_127_ residues with the neutral charged alanine residues. This mutant virus (DUL96C) manifested a smaller plaque size and significantly reduced growth rate compared to the wild-type virus (Towne-BAC) (data not shown). The mutant virus was serially passaged in fibroblasts until a bigger plaque size and an approximately 10,000-fold increased virus growth rate was achieved (data not shown). To define the mutations that accumulated during passaging of DUL96C virus, which could account for a compensatory effect on virus growth, we sequenced the genome of the passaged virus (UL96P10) and compared it to the Towne-BAC genome sequence (KF493877) (7). Although no major insertions, deletions, or substitutions were noticed in the UL96P10 genome, a total of 17 single-base substitutions were discovered.

For the purpose of genome sequencing, individual virus plaques of UL96P10 virus were purified and grown into virus stocks. Viral DNA was purified from pelleted virions from the cleared cell culture media using a DNeasy tissue kit (Qiagen, Inc.). The quantity and quality of purified dsDNA were determined using a Qubit fluorometric assay (Invitrogen). A Nextera XT DNA sample preparation kit (Illumina) was used to prepare the multiplexed paired-end libraries (2 × 150 bp) before sequencing using an Illumina MiSeq. The fastq files were imported into CLC Genomics workbench 6.0. We trimmed the reads for quality by using a limit of 0.05 (*P* of error, equivalent to Q13), using a maximum number of ambiguities of 2, and discarding reads of <15 bp. The *de novo* assembly used a word size of 45, a bubble size of 90, and a minimum contig length of 1,000 bp. Paired distances were autodetected and reads were mapped back onto contigs. All other parameters were default. A total of 21.2 million paired-end reads were collected. The number of scaffolds, scaffold *N*_50_, and total sequence length were 4, 49, and 232 kb, respectively. Each scaffold from each virus had an average coverage of >7,000. Gaps in the genome sequence were filled by PCR and sequencing of resultant amplicons. Open reading frames were identified using an NCBI-BLAST search and subsequently annotated in Sequin 12.30.

The 232,948-bp Towne-BAC_UL96_Mutant genome revealed a total of 17 single-base substitutions compared to the reference genome (KF493877), excluding the myc epitope tag engineered at the N terminus of UL96. The differences were located in the following regions of the genome: UL96, RNA4.9, upstream of TRS1, upstream of RNA4.9, downstream of UL97, and downstream of UL145.

### Nucleotide sequence accession number.

This whole-genome sequence of the Towne-BAC passaged virus has been deposited at GenBank under the accession no. KF493876.
